# Premature Burial

**Published:** 1910-07-09

**Authors:** 


*To the Editor of* The Hospital.

### PREMATURE BURIAL.

*To the Editor of* The Hospital.

Sir,—With reference to Mr. Jas. R. Williamson's letter on the subject of premature burial, I may say that I am acquainted with a lady who makes it her business to introduce the subject on every possible occasion, and by so doing she collects a vast amount of first-hand evidence from "all sorts and conditions of men."

Unfortunately, many people who have had personal experience themselves, or friends or relatives, of being nearly buried alive, are so extremely sensitive on the subject that they shrink from publicity.

But how valuable their experience would be to the cause in helping to disperse the deep-founded impression that when a person apparently ceases to breathe and is cold and stiff he must be dead.

An article in the *Nineteenth Century and After* written by Mr. Basil Tozer, dealing with the subject of premature burial, has the following quotation from the Paris *Figaro*, which some years ago investigated the possibility of people being buried alive :—

"The editor received over one hundred letters from different parts of France, all from persons who either had been almost buried alive or who knew of such cases. At first this may seem remarkable, but when we bear in mind that some twenty years ago it was reported officially that the proportion of bodies that come to life again in the French mortuaries is one in three hundred, we are less surprised. Indeed, Dr. Franz Hartmann himself has told us that during May and June 1896 he received no fewer than sixty-three letters from persons who had escaped premature burial through fortunate accident."

Nobody can depend upon "a fortunate accident" happening in every case of suspended animation. It is more probable that the "fortunate accident" does not occur at the critical moment, and that there are and have been many live burials of which the world knows nothing. It is a question whether, when a person is buried in a trance or in a state of suspended animation, that person dies in an unconscious state or becomes conscious on the return of animation.

Yours truly,

Live and Let Live.

THE HOSPITAL. July 9, 1910.

